# Nontraditional risk factors in chronic kidney disease: correlation between creatinine clearance, Framingham risk score, endothelial dysfunction, and inflammation

**DOI:** 10.1186/s43162-022-00110-2

**Published:** 2022-03-14

**Authors:** Ahmed Mohamed Tawfik, Heba Mohamed Tawfik

**Affiliations:** 1 Internal Medicine & Nephrology department, Faculty of Medicine, Ain-Shams University, Cairo, Egypt; 2 Geriatrics & Gerontology Department, Faculty of Medicine, Ain-Shams University, Cairo, Egypt

**Keywords:** Chronic kidney disease, Framingham risk, Carotid intimal medial thickness, Inflammation

## Abstract

**Background:**

Chronic kidney disease became a public health problem increasing healthcare burden. Our aim was to detect the relationship between cardiovascular risk, endothelial dysfunction, inflammation, and kidney function in chronic kidney disease patients and to detect the nontraditional factors affecting the decline in kidney functions.

**Methods:**

A cross-sectional study including 30 male and female patients with chronic kidney disease stages 3–5. Creatinine clearance and Framingham risk score points were calculated. Carotid intimal medial thickness was measured as well as absolute flow mediated dilatation in brachial artery. Highly sensitive C-reactive protein, parathyroid hormone, kidney function tests, and lipid profile were measured.

**Results:**

Framingham risk score points and carotid intimal medial thickness increased significantly with decreasing creatinine clearance (*p* 0.0025, 0.0285) respectively. A significant correlation was found between highly sensitive C-reactive protein and Framingham risk score points but not with carotid intimal medial thickness (*p* 0.0043, 0.2229) respectively. An inverse correlation was found between creatinine clearance and highly sensitive C-reactive protein (*p* 0.0174). Absolute flow mediated dilatation in brachial artery decreases with increasing Framingham risk score points and decreasing creatinine clearance (*p* 0.0044, 0.0269) respectively.

**Conclusion:**

There is correlation between chronic kidney disease and impaired vascular function, subclinical atherosclerosis, and heightened inflammatory response. Chronic kidney disease patients are at increased risk of cardiovascular events with higher [10-]year cardiovascular risk.

## Background

Chronic kidney disease (CKD) has shifted from the 36th cause of death in 1990 to the 19th cause in 2013 [1]. This represents a global healthcare burden due to in part its association with an increased risk of cardiovascular diseases (CVD) and cardiovascular events (CVEs) like angina pectoris and myocardial infarction. Even patients with lesser degrees of renal dysfunction are predisposed to such complications and some studies suggest that mild-to-moderate renal insufficiency can predict cardiovascular mortality. CKD is considered now by some researchers a powerful predictor of cardiovascular events [2, 3].

There is little information about the pathogenesis of cardiac diseases in CKD, especially in early stages and whether it correlates with the traditional risk factors such as dyslipidemia, diabetes, and hypertension. This is difficult to confirm because atherosclerosis, unless became severe, is often asymptomatic; hence, a direct examination of vessel wall is necessary to detect early atherosclerosis [4, 5]. Carotid artery intimal–medial thickness (CIMT) is a well-established index of systemic atherosclerosis and carotid artery disease is very often present concurrently with coronary artery disease and cerebrovascular strokes [6]. It was found that vascular calcification, increased carotid intima media thickness (CIMT), and the presence of carotid plaques are strongly associated with cardiovascular disease in CKD patients [7].

Framingham risk score (FRS) is the most widely used score to predict the risk of developing CVEs in 10 years. It was first developed in 1988 [8] to predict fatal and non-fatal myocardial infarction and angina pectoris and was validated in various ethnic groups in 2001 [9]. After revision in 2002 and in 2008 [10]; it became also validated to predict the risk of cerebrovascular strokes or transient ischemic attack, intermittent claudication, and also heart failure. However, it was noticed that CKD patients have other non-traditional risk factors which can increase the risk of CVEs. Some researchers reported that FRS had poor accuracy in predicting CVEs in CKD patients and that prediction is better with inclusion of other markers like C-reactive protein, coronary artery calcium score, carotid intima media thickness, and pulse wave velocity [11].

There is conflicting data regarding association between inflammation and the progression of CKD. In the Cardiovascular Health Study (CHS), higher levels of C- reactive protein (CRP), white blood cell count, and coagulation factors were associated with increasing creatinine levels [12]. However, a subsequent study using cystatin C as a measure of kidney function found no association between these biomarkers and decline in kidney function in the same population [13].

Recently, it was discovered that measurement of flow-mediated vasodilation (FMD) is considered an index of endothelium-dependent vasodilation, and when measured in the brachial artery using high-resolution ultrasound, it can be used as a method for assessing vascular function [14]. Endothelial function is initially impaired in the pathogenesis of atherosclerosis even before alteration in the vessel wall structure [15]. Measurement of FMD is noninvasive and reflects NO production, and there is also growing evidence that by FMD assessing endothelial function can serve as an independent predictor of cardiovascular events [16]. We hypothesize in this study that vascular function is impaired in CKD patients.

## Methods

Informed consent was obtained from all participants at the start of the study.

### Study design

A cross-sectional study was conducted to detect the relationship between cardiovascular risk, kidney function, and inflammation in CKD patients, to detect impairment in vascular function in such patients, and to detect the nontraditional factors affecting the decline in kidney functions such as inflammation, endothelial dysfunction, and CKD-mineral and bone disorder (CKD-MBD).

### Study population

Thirty male and female patients with CKD stages 3, 4, and 5 (not on dialysis) were included after accepting to participate in the study. They were admitted to the Nephrology Department at Ain-Shams University hospitals during the period between August and November 2018. Patients were diagnosed to have CKD if they had evidence of abnormalities of kidney structure or function lasting for more than 3 months and were classified into CKD stage 3, 4, and 5, based on estimated glomerular filtration rate (eGFR) level (mL/min/1.73 m^2^) of 30 to 59, 15 to 29, and 15 respectively according to the Cockcroft-Gault equation [17]:

$$\mathrm{Creatinine}\ \mathrm{clearance}\ \mathrm{in}\ \mathrm{men}=\left(140-\mathrm{age}\right)\times \mathrm{body}\ \mathrm{weight}/72\times \mathrm{serum}\ \mathrm{creatinine}$$

$$\mathrm{Creatinine}\ \mathrm{clearance}\ \mathrm{in}\ \mathrm{women}=\left[\left(140-\mathrm{age}\right)\times \mathrm{body}\ \mathrm{weight}/72\times \mathrm{serum}\ \mathrm{creatinine}\right]\times 0.85$$

### Exclusion criteria

Smokers and patients with diabetes mellitus were excluded from the study due to their strong association with inflammation and atherosclerosis and obese patients with body mass index ≥ 30 due to strong association with atherosclerosis. Also, patients having active infection and malignancy were excluded.

### Measures

After collection of demographic data from all participants, FRS was calculated [9], and points were recorded to detect 10-year risk of cardiovascular diseases. It takes into consideration the following factors: age, gender, systolic blood pressure value, whether or not the patient takes anti-hypertensive medications, the presence or absence of diabetes mellitus, smoking, high-density lipoproteins cholesterol (HDL-c), and total cholesterol values.

CIMT was measured as a measure of subclinical atherosclerosis and a predictor of cardiovascular events. B-mode ultrasonography was performed with vascular ultrasound system. Patients were examined in the supine position with the head tilted backwards. Intimal–medial thickness was measured in mm and was defined as the distance between the leading edge of the first echogenic line (lumen–intima interface) and the second echogenic line (media–adventitia interface) of the far wall. Three measurements were taken at 0.5, 1, and 2 cm below the carotid bifurcation of the common carotid artery on each side, and their arithmetic averages were calculated. The intimal–medial thickness of both sides (right and left) was also calculated and the average of these two values was calculated. All the CIMT measurements were performed by an experienced radiologist who was blinded to the clinical data.

Before measurement, each participant was fasting for at least 6 h and rested for 10–20 min in a quiet, temperature-controlled room. B-mode ultrasound image of the brachial artery in the dominant arm was obtained with the arm supinated and abducted about 80°. The diameter of the brachial artery was measured about 1–3 cm above the ante-cubital fossa in longitudinal plane. A cuff was placed at the forearm, 5–10 cm below the scan area. First brachial artery diameter was measured throughout the cardiac cycle at rest (baseline) according to the published guidelines [18]. It was measured three times and the average of these three measurements was calculated. The cuff around the forearm was then inflated 50 mmHg above the systolic blood pressure for 5 min with the occurrence of reactive hyperemia. Measures for 10-s interval 1 min after cuff release, 2 min after cuff release, or 3 min after cuff release with the highest post-occlusive measure were selected for comparison with baseline. Absolute brachial artery dilatation (absolute FMD) was calculated as the difference between the maximum diameter post-occlusion and the average baseline diameter.

Blood samples were obtained by venipuncture for measurement of serum creatinine, blood urea nitrogen, calcium (Ca), phosphorus (P), and intact parathyroid hormone (iPTH) levels. Lipid profile required 12 h of fasting. Blood samples were assayed within 24 h. Highly sensitive CRP (Hs CRP) was based on the principle of a solid phase ELISA. Kits were stored at 2–8 °C. All reagents were allowed to reach room temperature (18–22 °C) before use. Patient serum was diluted 100-fold prior to use. Obtained values of Hs CRP were in ng/ml. Patient samples with CRP concentrations greater than 10000 ng/ml were diluted 10-fold after the initial 100-fold dilution (total dilution 1:1000). Expected values of Hs CRP were from 68 to 8200 ng/ml.

### Statistical analysis

Analysis of data was carried out by using the 17th version of SPSS (SPSS, Chicago, IL, USA). Data is shown as the mean (*M*) and standard deviation (*SD*) for all quantitative variables. The frequency and percentage for qualitative variables was calculated. Correlation coefficients to find linear relationships between different variables were calculated using *r*-test or Spearman’s correlation coefficient. Values for *p* less than 0.05 were considered significant and values less than 0.001 were considered highly significant.

## Results

The mean age of the study population was 58 as shown in Table 1. Nineteen males and 11 females were included in the study. Fifty-three percent of the study participants were hypertensive. The mean value of FRS points was 10.83 (± 6.10) and for CIMT was 1.10 (± 0.20). Other laboratory data are detailed below. Etiology of CKD is described in Table 2. HTN was the cause in more than 50%, obstructive uropathy in more than 15%, and systemic lupus erythematosus in 10% of the study population. Other causes were chronic glomerulonephritis, obstructive uropathy, and recurrent urinary tract infection. Table 3 describes factors correlated with FRS points. There is a highly significant correlation between FRS points and CIMT (*p* 0.0007), a significant inverse correlation between FRS points and both creatinine clearance and absolute FMD (*p* 0.0025, 0.0044) respectively. FRS points increased significantly with increasing Hs CRP levels (*p* 0.0043). Regarding lipid profile, there is a highly significant correlation between FRS points and serum triglyceride levels and no significant correlations between FRS points and other laboratory results. Regarding CIMT in Table 4, there is a highly significant correlation between CIMT and age (*p* 0.0000), a significant correlation between CIMT and serum cholesterol level (*p* 0.0100), and an inverse correlation between CIMT and creatinine clearance (*p* 0.0285); otherwise, no other significant correlations were found. Table 5 shows inverse correlation between creatinine clearance and Hs CRP (*p* 0.0174) and with FRS and CIMT as mentioned before. Absolute FMD decreased with decreasing creatinine clearance (*p* 0.0269).

**Table 1:** Demography and laboratory data of the study population

	Mean ± SD
Age (years)	58 ± 13.96
Gender	
Male	19 (63%)
Female	11 (37%)
Number of hypertensive (%)	16 (53%)
Framingham risk score (%) Mean/SD	8.22 ± 7
Framingham risk score points Mean/SD	10.83 ± 6.10
BUN (mg/dl) Mean/SD	58.97 ± 11.58
Creatinine (mg/dl) Mean/SD	5.52 ± 1.01
Calcium (mg/dl) Mean/SD	8.73 ± 0.67
P (mg/dl) Mean/SD	5.54 ± 1.03
iPTH (Pg/ml) Mean/SD	255.56 ± 125.61
Total cholesterol (mg/dl) Mean/SD	152.17 ± 34.79
Triglycerides (mg/dl)	147.63 ± 53.11
HDL (mg/dl)	52.86 ± 17.32
LDL (mg/dl)	136.37 ± 33.18
Intimal medial thickness (mm) Mean/SD	1.10 ± 0.20
Absolute brachial artery dilatation (absolute FMD) (mm)	0.6 ± 0.43
High sensitivity c-reactive protein (Hs CRP) (ng/ml)	5778.33 ± 2285.93

*SD* standard deviation, *BUN* blood urea nitrogen, *P* phosphorus, *iPTH* intact parathyroid hormone, *LDL* low-density lipoprotein, *HDL* high-density lipoprotein, *FMD* flow-mediated dilatation, *Hs CRP* high-sensitivity c-reactive protein, *mg/dl* milligram/deciliter, *Pg/ml* picogram/milliliter, *mm* millimeter, *ng/ml* nanogram/milliliter

**Table 2:** Etiology of CKD in the study population

Etiology of CKD	Number/percent (%)
HTN	16
	53.3%
Systemic lupus erythematosus	3
	10%
Obstructive uropathy	5
	16.7%
Chronic glomerulonephritis	2
	6.7%
Reflux nephropathy	2
	6.7%
Recurrent UTI	2
	6.7%
Total	30
	100.0%

*CKD* chronic kidney disease, *HTN* hypertension, *UTI* urinary tract infection

**Table 3:** Correlation between Framingham risk score, creatinine clearance, inflammation, and laboratory data in CKD

	FRS points ***r*** Pearson’s correlation	***p*** value
IMT	0.586	**0.0007**
Creatinine clearance	− 0.532	**0.0025**
hs CRP	0.506	**0.0043**
Ca x p product	0.152	0.4239
Ca level	0.047	0.8068
iPTH	0.308	0.0974
Bun level	0.242	0.1978
Triglycerides	0.592	**0.0006**
LDL level	0.219	0.2449
Absolute brachial artery dilatation (absolute FMD) (mm)	− 0.505	**0.0044**

*CKD* chronic kidney disease, *FRS* Framingham risk score, *IMT* intimal medial thickness, *Hs CRP* high-sensitivity c-reactive protein, *Ca* calcium, *P* phosphorus, *iPTH* intact parathyroid hormone, *BUN* blood urea nitrogen, *LDL* low-density lipoprotein, *FMD* flow-mediated dilatation mm millimeter

**Table 4:** Correlation between IMT, age, inflammation and laboratory data in CKD

	IMT ***r*** Pearson correlation	***p*** value
Age	0.692	**0.0000**
Creatinine Clearance	− 0.400	**0.0285**
hs CRP	0.229	0.2229
Ca x p product	0.249	0.1847
iPTH	0.021	0.9127
Bun level	0.114	0.5483
Total cholesterol	0.463	**0.0100**
Triglycerides	0.125	0.5104
HDL level	− 0.088	0.6476
LDL level	0.145	0.4439
Absolute brachial artery dilatation (absolute FMD) (mm)	− 0.344	0.0627

*IMT* intimal medial thickness, *CKD* chronic kidney disease, *Hs CRP* high-sensitivity c-reactive protein, *Ca* calcium, *P* phosphorus, *iPTH* intact parathyroid hormone, *BUN* blood urea nitrogen, *HDL* high-density lipoprotein, *LDL* low-density lipoprotein, *FMD* flow-mediated dilatation mm millimeter

**Table 5:** Correlation between creatinine clearance, cardiovascular risk, laboratory and endothelial dysfunction measures

	Creatinine clearance ***r*** Pearson correlation	***p*** value
FRS	− 0.532	**0.0025**
IMT	− 0.400	**0.0285**
Ca x p product	− 0.127	0.5070
Ca level	0.189	0.3198
P level	− 0.175	0.3412
iPTH	− 0.315	0.0900
Bun level	− 0.193	0.3146
Total cholesterol	− 0.263	0.1603
Triglycerides	− 0.131	0.4902
HDL level	0.296	0.1124
LDL level	0.079	0.6766
hs CRP level	− 0.432	**0.0174**
Absolute brachial FMD (mm)	0.404	**0.0269**

*FRS* Framingham risk score, *IMT* intimal medial thickness, *Ca* calcium, *P* phosphorus, *iPTH* intact parathyroid hormone, *BUN* blood urea nitrogen, *HDL* high-density lipoprotein, *LDL* low-density lipoprotein, *Hs CRP* high-sensitivity c-reactive protein, *FMD* flow-mediated dilatation *mm* millimeter

## Discussion

CKD was found to be a powerful predictor of CVEs. Although the association between decline in kidney function and CVD risk is not fully understood up till now, there are some explanations. The high prevalence of traditional (such as age, sex, smoking, diabetes mellitus, hypertension, dyslipidemia) and non-traditional (such as declining eGFR, proteinuria, anemia, inflammation, endothelial dysfunction, and CKD-MBD) cardiovascular (CV) risk factors are the major accused factors in the pathogenesis [3, 13, 19]. Barzouhi et al. [20] reported that patients with CKD appear to have a higher risk of CVD, independent of traditional cardiovascular risk factors. We measured the relation between age and dyslipidemia as traditional risk factors and CV risk, but we concentrated on the non-traditional risk factors such as creatinine clearance, oxidative stress, inflammation, endothelial dysfunction, calcium phosphorus product, and secondary hyperparathyroidism.

Both CIMT and FRS points showed inverse correlation with creatinine clearance. The REACTION Study [21] in China found that even mildly reduced eGFR (60–89 mL/min per 1.73 m^2^) was associated with an increased 10-year Framingham risk for coronary artery disease. Preston et al. [22] reported more severe atherosclerosis with higher values of CIMT in patients with CKD stages 3 and 4 when compared with healthy participants. Zhang et al. [23] found a significant increase in CIMT in patients with stage 2–3 CKD and concluded that arterial changes are not a stigma of advanced kidney disease and might occur earlier in the course of CKD than previously believed. CIMT was found to increase with increasing age of patients with CKD.

A strong inverse correlation was found between FRS points and FMD as a marker of endothelial dysfunction in CKD patients; however, that correlation between FMD and CIMT was insignificant. In the Multi-Ethnic Study of Atherosclerosis, incorporating > 3000 participants, FMD was found to predict future CVEs. In addition, FMD in combination with the FRS helped to classify CV risk better than FMD or the Framingham risk score alone [24]. There are conflicting results regarding the clinical impact of endothelial function on intimal-medial thickness. Weidinger et al. [25] did not find in their study on male patients undergoing coronary angiography a correlation between brachial IMT and brachial FMD.

Hs CRP correlated with FRS points which agreed with Albert and colleagues [26] who conducted the study on > 1500 middle-aged men and women participants and found that participants in the lowest cardiovascular risk category had CRP levels that were half or more of those levels of participants in the highest risk category and considered Hs CRP as a marker in the prediction of cardiovascular risk. Hs CRP can bind to damaged endothelial cells and aggregate low-density lipoprotein with stimulation of tissue factor production, increasing CVEs. Hs CRP also showed inverse association with creatinine clearance. Elevated levels were found not only in hemodialysis patients but also in elderly persons with renal insufficiency, and it is postulated that it may contribute to the pathogenesis of glomerulosclerosis through deposition along the walls of glomerular capillaries [27].

Impairment in vascular function was found in CKD patients as predicted by brachial FMD as shown by Kopel et al. [28] who demonstrated that impairment in vascular endothelial function in patients with advanced CKD is substantial and greater than that observed in individuals with clinical vascular disease but good kidney function.

## Conclusion

CKD is correlated with impaired vascular function, subclinical atherosclerosis, and heightened inflammatory response. CKD patients are at increased risk of CVEs with higher 10-year CV risk especially in late stages of the disease.

### Limitation of the study

The study was conducted on small sample size. Larger samples are needed for better generalization of results and inclusion of patients on hemodialysis. Urinary protein excretion was not performed, which is a well-known risk factor for vascular diseases and atherosclerosis and the study concentrated only on non-traditional risk factors. No longitudinal follow-up for the patients was performed, and we used CG formula to assess kidney functions which measures creatinine clearance (not GFR) which becomes less accurate as the kidney function worsens. We could not exclude age as a confounder in risk factors as it is a part of FRS and of CG formula as well.

## Data Availability

Available upon request.

## Abbreviations

CKD: Chronic kidney disease
CVD: Cardiovascular diseases
CVEs: Cardiovascular events
CIMT: Carotid artery intimal–medial thickness
FRS: Framingham risk score
CHS: Cardiovascular Health Study
CRP: C-reactive protein
FMD: Flow-mediated vasodilation
CKD-MBD: CKD-mineral and bone disorder
NKF: National Kidney Foundation
K/DOQI: Kidney Disease Outcomes Quality Initiative
eGFR: Estimated glomerular filtration rate
HDL-c: High-density lipoproteins cholesterol
Ca: Calcium
P: Phosphorus
iPTH: Intact parathyroid hormone
HS CRP: Highly sensitive CRP
M: Mean
SD: Standard deviation
